# Psychrophilic Quorum Sensing Genes Enable Unimodal, Adjustable Protein Expression Across the Entire *Escherichia coli* Population

**DOI:** 10.3390/biotech15030057

**Published:** 2026-07-21

**Authors:** Ekaterina Scheglova, Sabina Nebieva, Kamilla Mekhantseva, Siarhei Bukhalovich, Anna Kudryavtseva, Nikolay Bondarev, Nikolay Ilyinsky, Sergey Bazhenov, Ilya Manukhov

**Affiliations:** Moscow Center for Advanced Studies, Kulakova Str. 20, 123592 Moscow, Russia; scheglova.ekatserg@gmail.com (E.S.); bazhenov1994@gmail.com (S.B.)

**Keywords:** *Escherichia coli*, *Aliivibrio logei*, expression system, quorum sensing, LuxR, LuxI, flow cytometry

## Abstract

A previous study demonstrated that quorum sensing (QS) genes from the psychrophilic bacterium *Aliivibrio logei* can be used in *Escherichia coli* to obtain bacterial cultures with controlled amounts of a target protein. However, the QS system may be induced non-uniformly across the cell population. In this study, we used an expression vector containing *luxR/luxI* regulatory genes from *A. logei* carrying *sfGFP* as a reporter gene. Reporter expression was regulated by the autoinducer 3OxoC6-HSL, activated at 22 °C, and terminated at 37 °C. Flow cytometry was used to assess the GFP fluorescence distribution at the single-cell level. The system provided dose-dependent unimodal expression lacking formation of distinct ON/OFF subpopulations, while the robust coefficient of variation decreased with increasing autoinducer concentration. The autoinducer synthase LuxI enabled autoinduction, but under the tested conditions, no substantial effect on expression homogeneity was detected. Raising the temperature to 37 °C effectively halted expression, allowing intermediate target protein values to be fixed at the single-cell level. Overall, the developed system represents a promising tool for biotechnological applications requiring precise and uniform control of expression.

## 1. Introduction

Bacterial expression of recombinant proteins plays an important role not only in their production but also in their study. Among the most widely used inducible expression systems based on *Escherichia coli* are the IPTG/lactose-inducible P*lac*, P*tac*, P*trc*, and PT7 promoters, the arabinose-inducible P*araBAD*, and the rhamnose-inducible P*rha* [1,2,3].

Despite their popularity and high expression efficiency, some widely used inducible systems exhibit significant expression cell-to-cell heterogeneity. In particular, T7-*lac*-type systems can generate bimodal distributions of target protein expression, characterized by the presence of fully induced (ON) and non-induced (OFF) cell subpopulations [4,5]. This heterogeneity is associated with a positive feedback loop arising from LacY-mediated inducer transport. To increase the homogeneity of such systems, *lacY*^−^ strains lacking active inducer transport into the cell are often used. In biotechnological applications, the presence of subpopulations with different expression states may reduce overall culture productivity, increase metabolic load, and complicate process scaling.

Recently, expression vectors based on quorum sensing (QS) modules have been developed. QS is the ability of bacteria to coordinately regulate gene expression depending on population density and is used across bacterial species to control a wide range of functions, including bioluminescence, virulence factor production, biofilm formation, sporulation, competence, and antibiotic biosynthesis [6,7]. Marine mesophilic bacteria *Aliivibriofischeri* and psychrophilic *Aliivibrio logei* regulate luminescence using a QS circuit [7,8,9]. In these bacteria, the synthetase LuxI produces a set of acyl-homoserine lactones (AHLs) called autoinducers (AIs), of which L-homoserine N-(3-oxohexanoyl)-lactone (3OxoC6-HSL) is the main one [10]. When the threshold concentration of AIs in the medium is reached, and, consequently, when the threshold population density is reached, LuxR receptor proteins bind to AIs, attach to DNA in this form at a site called the *lux*-box, and induce the expression of genes responsible for luminescence [11]. QS-based expression systems provide the possibility of autoinduction through endogenous autoinducer synthesis and, at the same time, enable regulation of expression levels by adding exogenous autoinducer.

A number of expression vectors based on *luxR/luxI* genes from *A. fischeri* have been constructed for use in *E. coli* and *Bacillus subtilis* [12,13,14]. The primary rationale for creating such systems was the use of an inducer not associated with sugar metabolism and the possibility of autoinduction determined by the density of the culture and not by the composition of the medium. Genetic modifications and the use of different promoters upstream of the *luxR* and *luxI* genes made it possible to shift the moment of autoinduction; however, in some cases, such modifications radically changed the kinetics of induction, making the system more similar to a constitutively functioning one [15]. AHLs passively penetrate the membrane, which should promote uniformity of induction. However, in response to the appearance of an AI in the medium, the cell itself begins to produce more AI [16]. In natural bacterial strains, marked heterogeneity and bimodality in the induction of QS systems was observed. For example, it has been shown that individual cells of *A.fischeri* differ markedly in brightness even at high AI concentrations and after prolonged induction [17]. The uniformity of induction of expression systems based on QS modules and *E. coli* cells remains unstudied.

Based on LuxR2/LuxI regulatory elements from *A. logei*, we constructed an expression system which enables target protein expression in an AI-dose-dependent manner upon temperature reduction to 22 °C, while raising the temperature to 37 °C interrupts induction [18]. Studying protein function in vivo requires controlled production of the protein of interest without compromising cell viability. However, overexpression of many proteins can perturb cellular metabolism, suppress growth, and ultimately result in cell death [19,20,21]. The ability both to tune the expression level via exogenous AI concentration and to terminate synthesis on demand via a temperature shift is therefore directly relevant for such applications, motivating a quantitative, single-cell-resolution characterization of how uniformly and predictably this system behaves across the induced population.

While our previous work [18] characterized AI-dose-dependent, temperature-switchable regulation of a LuxR2/LuxI-based expression system at the population level, it remained unknown whether individual cells within an induced population respond uniformly or whether the population instead comprises distinct ON/OFF subpopulations. 

In this study, we further modified this expression vector by shortening the non-coding region between the transcription and translation start sites (from 297 bp to 66 bp) and replacing the “−10” promoter element with the consensus TATAAT sequence and used the resulting construct (pBMS10) with *sfGFP* gene insertion to resolve expression at single-cell resolution by flow cytometry. We additionally introduce a *luxI*-deletion variant (pBMS10ΔI) to test the effect of a positive feedback loop of the QS module on the homogeneity of target gene expression.

## 2. Materials and Methods

### 2.1. Strains and Plasmids

All experiments were conducted with *E. coli* TG1 strain transformed with pBMS10-sfGFP or pBMS10ΔI-sfGFP plasmids. pBMS10-sfGFP is a derivative of the pIR-DPAl expression vector [18] with insertion of *sfGFP* as a target gene and modified promoter region upstream of it (shortened distance between transcription and translation start sites from 297 bp to 66 bp and change of “−10” sequence to the consensus TATAAT). pBMS10ΔI-sfGFP is a derivative of pBMS10-sfGFP with a deleted *luxI* gene.

### 2.2. Culture Medium and Growth Conditions

*E. coli* cell cultures were grown at 37 °C in lysogeny broth (LB). The LB medium was composed of 1% tryptone, 0.5% yeast extract, and 1% NaCl and supplemented with 50 μg/mL kanamycin. To obtain solid medium, agar was added to a final concentration of 1.5%. Culture media components were purchased from Dia-M (Moscow, Russia).

### 2.3. Molecular Cloning

Plasmid pBMS10 was obtained by circular PCR using primers TATAGT-Rev and PluxCshort-Dir on the pIR-DPAl matrix [15], followed by self-ligation via the Gibson assembly method [22]. Plasmid pBMS10-sfGFP was obtained by Gibson assembly of two DNA fragments: the NdeI-linearized vector pBMS10 and the *sfGFP* gene amplified using primers sfGFPdir-pMBS and sfGFPrev-pMBS. Deletion of *luxI* was performed by PCR using primers pBMS-DluxIrev and pBMS-DluxIdir on the pBMS10-sfGFP matrix, followed by self-ligation via the Gibson method. The resulting plasmid was named pBMS10ΔI-sfGFP.

The T7-phage polymerase gene was cloned from *E. coli* BL21(DE3) Gold gDNA using primers T7_pIRDAl_dir and T7_pIRDPAl_rev. The 2700 bp PCR product was inserted into the Bsp13I-linearized vector via Gibson assembly. Primer sequences are provided in Table S1.

PCR reactions were performed using high fidelity DNA polymerase Q5 from NEB (Ipswich, MA, USA). The assembly of genetic constructs according to the Gibson Assembly method was carried out using NEB enzymes (Ipswich, MA, USA). NdeI endonuclease was obtained from SibEnzyme (Novosibirsk, Russia).

### 2.4. Sample Preparation

*E. coli* TG1 pBMS10-sfGFP and *E. coli* TG1 pBMS10ΔI-sfGFP cell cultures were grown to OD ≈ 0.5 at 37 °C, 200 rpm in liquid LB medium in a volume of 12 mL. 3OxoC6-HSL was selected as the inducer because it is the main AI of the LuxI-LuxR system of the most closely related bacterial species of *A. logei—A. fischeri*. 3OxoC6-HSL was obtained from SigmaAldrich (St. Louis, MO, USA). The cultures were divided into 4 parts of 3 mL each, 3OxoC6-HSL was added to a concentration of 0 nM, 10 nM, 10 μM (in 2 tubes), and incubated at 22 °C, 200 rpm. After 30 min and 3 h of incubation, samples were collected from the 0 nM, 10 nM, and 10 μM 3OxoC6-HSL cultures to assess sfGFP expression. One sample containing 10 μM 3OxoC6-HSL, after sample collection at 30 min of incubation at 22 °C, was transferred for further growth at 37 °C for 2.5 h, followed by an additional sample collection.

Fixation of the obtained samples for measuring fluorescence at the level of individual cells was performed by treatment with 2% paraformaldehyde: 1 mL of culture was centrifuged, the pellet was resuspended in 1 mL of PBS, 1 mL of culture was centrifuged, the pellet was resuspended in 200 μL of PBS + 2% paraformaldehyde and incubated for 15 min at room temperature, and the pellet was resuspended in 200 μL of PBS. All centrifugations were performed for 1 min at 10,000× *g*.

### 2.5. Fluorescence Intensity Measurements

The integral fluorescence of the cultures and OD600 were measured using a Synergy HT instrument (Biotek, Winooski, VT, USA) with excitation at 480 nm and emission detection at 515 nm.

Cell fluorescence was measured using an Attune NxT flow cytometer (Invitrogen, Waltham, MA, USA). For flow cytometric analysis, cell suspensions were fixed in 2% PFA according to the protocol described above. Initial gating was performed using a density plot of FSC-H versus SSC-H, after which cellular fluorescence was analyzed in the BL1-H channel (488 nm excitation, 530/30 nm emission filter; hereafter referred to as GFP-H).

Microscopic measurements were performed using an LSM780 inverted confocal fluorescence microscope (Carl Zeiss, Oberkochen, Germany). Frames were acquired at a resolution of 1024 × 1024 pixels in channel mode with a detection range of 490–540 nm, with excitation by an argon laser at 488 nm (LASOS, Jena, Germany), with a total per-frame exposure of approximately 30 s. A 63× oil immersion objective with a numerical aperture of 1.4 was used. Frame size: 224.92 × 224.92 μm. Simultaneously with the fluorescence channel mode, bright-field imaging was performed in T-PMT mode to visualize all cells in the field. For analysis of random signals, the fluorescence spectrum of cells was analyzed in λ-mode using a 34-channel QUASAR detector (Leica Microsystems, Wetzlar, Germany).

### 2.6. Statistical Analysis

Flow cytometry data were processed using Python 3 with the readfcs, numpy, scipy, and plotly packages. The data were gated based on FSC-A (10^4^–2 × 10^5^) and SSC-A (1.5 × 10^3^–10^5^) parameters. The fluorescence intensity of sfGFP from the GFP-H channel was used for subsequent analysis.

Visualization of fluorescence distributions was performed using kernel density estimation (KDE) with a Gaussian kernel. The kernel bandwidth was set as kde.factor × 0.75 to achieve an optimal balance between smoothing and preservation of distribution details. KDE values were normalized to the maximum.

To quantify expression heterogeneity, the robust relative coefficient of variation (rCV) on the raw non-logarithmic data after gating was calculated [23]. The median was used as the measure of central tendency, and the median absolute deviation (MAD) was used as the measure of dispersion:

rCV = (1.4826 × MAD)/median,(1)

This approach is robust to asymmetric distributions and the presence of outliers, which is typical of flow cytometry data.

To assess the statistical significance of differences in median fluorescence intensity and rCV between experimental conditions, a two-step statistical approach was applied. First, for the AI concentration- and time-dependent experiments, a factorial linear mixed-effects model was fitted separately to median log_10_-transformed fluorescence and to rCV as response variables, with presence of *luxI*, AI concentration, induction time, and their interactions as fixed effects and biological replicate as a random effect. For the interrupted-induction experiment, an analogous two-way linear mixed-effects model was fitted with vector type and induction condition as fixed effects. In both cases, the statistical significance of each fixed effect and interaction was assessed using Type III Wald F-tests with Kenward–Roger degrees of freedom. Second, for factors or interactions found to be statistically significant in the omnibus F-test, post hoc pairwise comparisons of estimated marginal means were performed, with *p*-values corrected for multiple testing using the Holm method. Statistical analyses were performed in R (v. 4.4.2) using the lme4, lmerTest, car, and emmeans packages. Significance levels are indicated in figures as follows: ns—not significant; * *p* < 0.05; ** *p* < 0.01; *** *p* < 0.001. Complete results of all factorial analyses and pairwise comparisons, including F-statistics, t-statistics, and exact *p*-values, are provided in the Supplementary Materials, Tables S2 and S3.

## 3. Results

### 3.1. Possibilities of Fixing the Amount of Target Protein in Cell Culture

**Figure 1:**
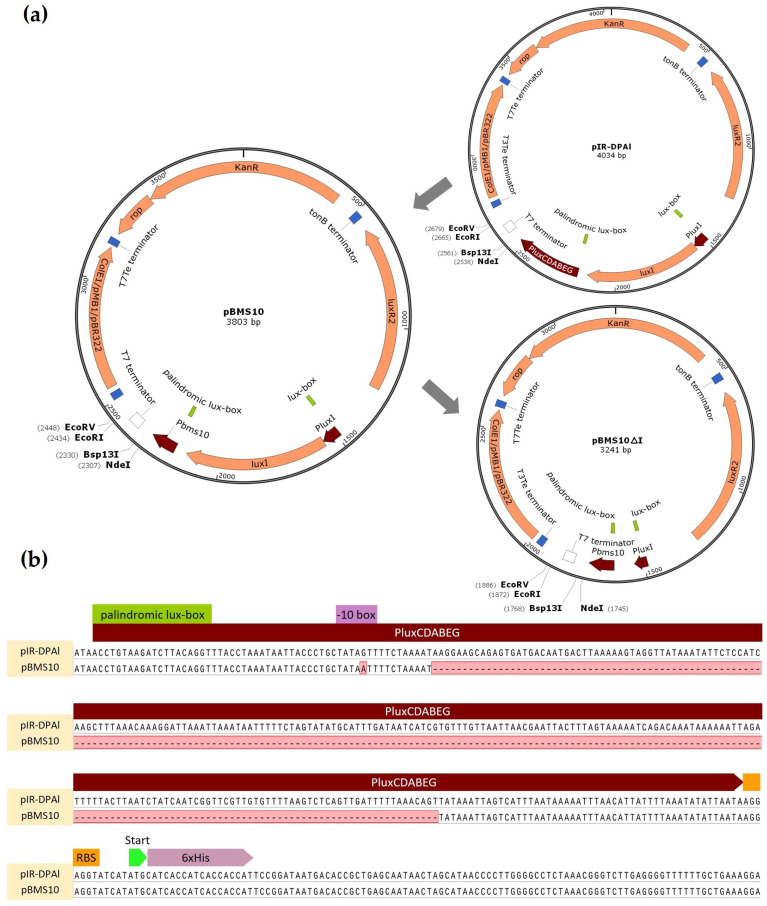
(**a**)—Schematic representation of the pBMS10, pIR-DPAl, and pBMS10ΔI. (**b**)—Comparison of promoter sequences of pIR-DPAl and new promoter in pBMS10 with a shortened 5′-UTR and a consensus −10 sequence.

During this study, a modified expression vector pBMS10 (Figure 1) was developed, based on the temperature-switchable vector pIR-DPAl. Expression of the target gene in this vector is also mediated by the *luxR2* gene; however, the target gene is placed under the control of a hybrid promoter based on P*_luxCDABEG_* from *A. logei*: with a shortened 5′-UTR, a fully palindromic lux-box (ACCTGTAAGATCTTACAGGT), and a consensus −10 sequence (TATAAT). To investigate the expression system activity and assess the homogeneity of induction, the *sfGFP* gene was inserted into the vector as the target gene. The presumed cause of the heterogeneity observed in QS system activity in *A. fischeri* cells is intracellular autoinducer synthesis and its non-uniform distribution across the medium and cells. Therefore, to investigate the effect of *luxI* gene activity on the homogeneity of induction, a plasmid lacking *luxI* was also constructed.

**Figure 2:**
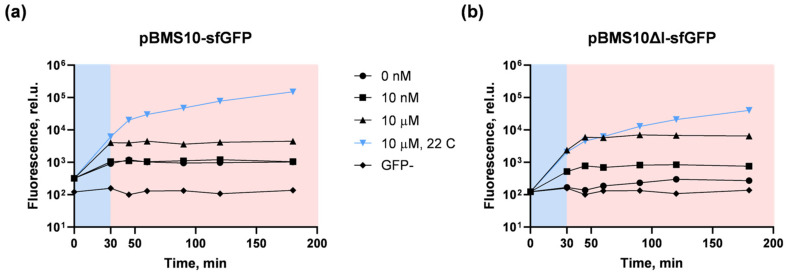
Total, culture-integrated sfGFP fluorescence (measured directly by plate reader, without normalization to culture density) of TG1 pBMS10-sfGFP (**a**) and TG1 pBMS10ΔI-sfGFP (**b**) cells upon a short-term decrease in temperature and the addition of AI to the medium. Black curves (circles, squares and triangles) represent samples induced by 0 nM, 10 nM and 10 μM AI, respectively, and by decreasing the temperature to 22 °C for 30 min (blue background), followed by interruption of induction by increasing the temperature to 37 °C (pink background). Data from the same cells with the addition of 10 μM AI, which continued to be incubated at 22 °C without raising the temperature, are shown as a control (blue curve). The autofluorescence background of wild-type TG1 cells lacking the sfGFP reporter, grown and measured under the same conditions, is shown as a control (diamonds).

To assess the functionality of the constructed plasmids, *sfGFP* expression was induced by reducing the temperature to 22 °C and by exogenous addition of 3OxoC6-HSL at concentrations of 10 nM (low induction) and 10 μM (maximal induction). After 30 min of incubation, the temperature was returned to 37 °C and incubation continued with periodic sampling to determine GFP accumulation. The total culture fluorescence measurements are shown in Figure 2.

It was shown that decreasing the temperature to 22 °C induced target protein expression in the presence of AI, while raising the temperature back to 37 °C interrupted induction. To confirm that the observed plateau in total fluorescence following the shift to 37 °C reflects cessation of sfGFP synthesis rather than growth arrest, OD600 growth curves for the TG1 pBMS10-sfGFP 10 μM AI condition (both with and without interruption of induction) are provided in Supplementary Figure S1, confirming that the culture continued to grow after the temperature shift. The fluorescence of TG1 pBMS10-sfGFP increases over time even without exogenous AI addition, indicating the system’s capacity for autoinduction due to LuxI activity. At this stage of autoinduction, the addition of 10 nM AI no longer has a significant effect. Deletion of *luxI* from the system abolishes autoinduction: fluorescence of TG1 pBMS10ΔI-sfGFP without AI addition remains at a background level. Target protein expression in both systems occurs in an AI-dose-dependent manner. However, due to accumulation of endogenous AI in TG1 pBMS10-sfGFP and autoinduction, the difference in fluorescence levels between cells treated with different concentrations of exogenous AI becomes less pronounced over time. In contrast, the system lacking *luxI* provides more precise control of expression by the external autoinducer.

To independently verify that the temperature shift to 37 °C interrupted de novo sfGFP synthesis, whole-cell lysates normalized by OD600 were analyzed by SDS–PAGE (Figure S2). SfGFP accumulated progressively during incubation at 22 °C in the presence of 10 μM AI. In contrast, after shifting the cultures to 37 °C, no further increase in the sfGFP band was observed. Instead, the relative intensity of the band gradually decreased, consistent with dilution of the previously synthesized protein during continued cell growth rather than ongoing expression. The decrease was most pronounced in cultures induced for only 30 min before the temperature shift, whereas cultures induced for 90 min retained a clearly detectable sfGFP band after the subsequent incubation at 37 °C. These results are fully consistent with the fluorescence measurements.

To evaluate the performance of the *A. logei* LuxR2/LuxI-based expression system using a non-fluorescent protein of biotechnological relevance, we placed the T7 RNA polymerase gene under the control of a LuxR2-regulated promoter. Induction experiments showed significant accumulation of T7 RNA polymerase when incubated at 22 °C for 19 h (Figure S3).

### 3.2. Intermediate Content of the Target Protein by Varying Exogenous AI Concentration

**Figure 3:**
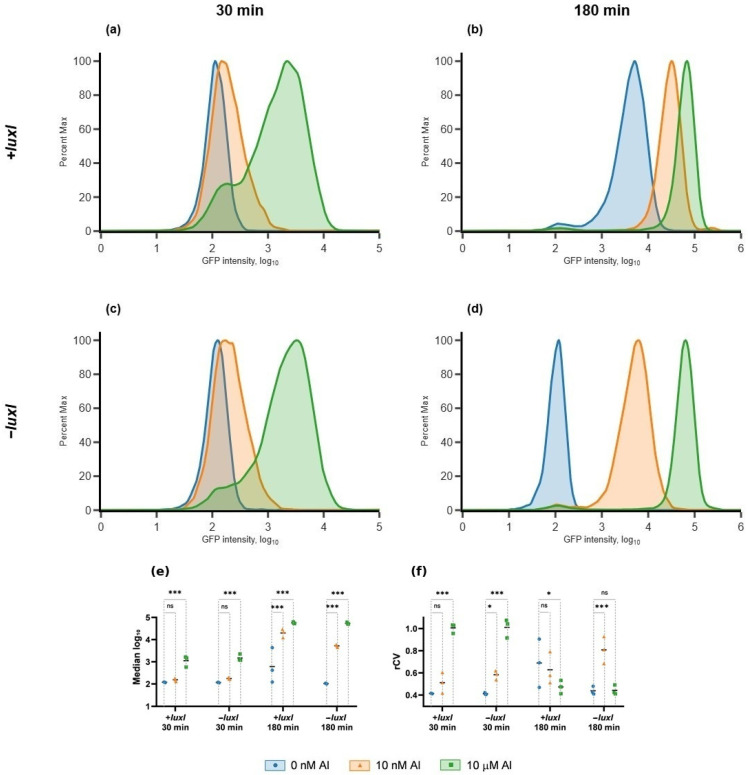
Distributions of sfGFP fluorescence in TG1 pBMS10-sfGFP (**a**,**b**) and TG1 pBMS10ΔI-sfGFP (**c**,**d**) cells at various autoinducer concentrations after 30 min (**a**,**c**) or 180 min (**b**,**d**) of induction. Panels (**e**,**f**) present quantitative summaries of three biological replicates, showing median fluorescence intensity and rCV, respectively. Asterisks denote statistical significance of pairwise comparisons: * *p* < 0.05; *** *p* < 0.001.

Theoretically, intermediate fluorescence values could be achieved through either incomplete promoter activation with uniform expression throughout the entire population or complete promoter activation in some cells resulting in a mixture of induced and non-induced cells. The aim of the following analysis was to determine which of these scenarios describes the behavior of the developed quorum-sensing-based expression system. To assess expression heterogeneity at the single-cell level, flow cytometry analysis was performed. *E. coli* TG1 pBMS10-sfGFP and *E. coli* TG1 pBMS10ΔI-sfGFP cells were grown to OD ~0.5 at 37 °C. The cell cultures were then divided into equal portions, 10 nM or 10 μM of the AI was added, and incubation continued at 22 °C. No exogenous AI was added to the control sample. After 30 and 180 min, cell samples were collected and the distribution of GFP content at the single-cell level was assessed (Figure 3). The experiment was performed in multiple biological replicates; the raw fluorescence intensity histograms are provided without smoothing in the Supplementary Materials (Figures S4 and S5). Complete results of pairwise comparisons are provided in the Supplementary Materials (Table S2).

A shift in the cellular fluorescence distribution is already observed after 30 min of induction with addition of 10 μM AI. At early induction time, the distributions remain relatively broad, and the rCV increases with increasing exogenous AI concentration. Additionally, at 10 μM AI, a subtle bimodality is observed, reflecting the initial phase of expression activation and possible stochastic variation in the process.

As induction time is extended to 180 min, the distributions become narrower and more clearly separated for different AI concentrations in the medium. No formation of cell subpopulations is observed at intermediate expression levels. Moreover, increasing the concentration of exogenous AI leads to a reduction in the width of the distribution.

The data in Figure 3e,f illustrate that the induction level and variation are repeated with high accuracy from experiment to experiment despite the relatively high rCV with 30 min of induction by 10 μM AI. In contrast, the 180 min of autoinduction by AI varies quite significantly across repeats.

Comparison of fluorescence distributions in the system with and without *luxI*, under the tested conditions and with the current number of biological replicates, showed no significant effect of the autoinducer synthase on expression homogeneity, while *luxI* determined the ability for autoinduction.

To visually confirm the homogeneity of expression at the individual cell level, fluorescence microscopy analysis of cultures induced by different concentrations of the AI was performed. Images of cells after 3 h of induction with the addition of 10 nM and 10 μM of the inducer are shown in Figure S6.

### 3.3. Intermediate Content of the Target Protein Due to Induction Interruption

**Figure 4:**
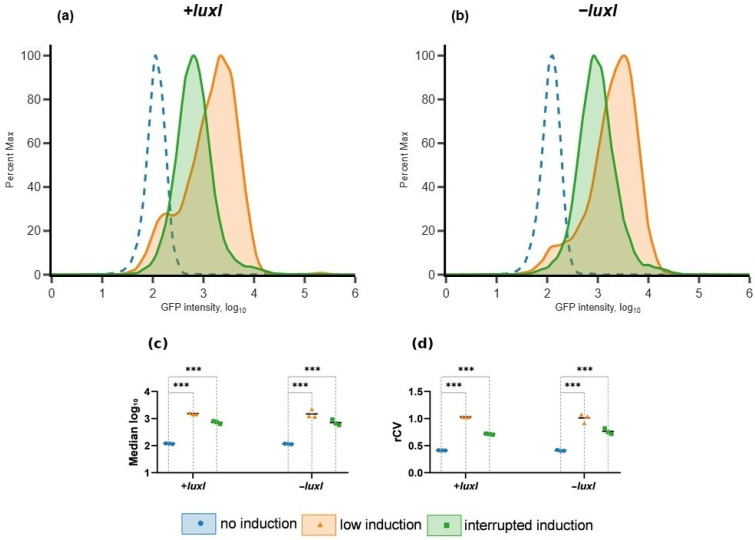
Comparison of sfGFP fluorescence distributions measured immediately after 30 min of induction (low induction) and after 30 min of induction with subsequent incubation at 37 °C for 2.5 h (interrupted induction) in TG1 pBMS10-sfGFP (**a**) and TG1 pBMS10ΔI-sfGFP (**b**) cells upon addition of 10 μM autoinducer. Cells without the addition of AI, immediately after 30-minute incubation at 22°C, were used as a control (no induction). Panels (**c**,**d**) present quantitative summaries of three biological replicates, showing median fluorescence intensity and rCV, respectively. Asterisks denote statistical significance of pairwise comparisons: *** *p* < 0.001.

The ability of the system to fix an intermediate amount of target protein in the cell upon interruption of induction was tested in systems induced by addition of 10 μM AI and incubation at 22 °C for 30 min, followed by stopping synthesis by increasing the temperature to 37 °C and subsequent incubation for 2.5 h. Fluorescence distributions at the single-cell level before and after interruption of induction are shown in Figure 4. Corresponding replicate distributions without smoothing are provided in the Supplementary Materials (Figure S5). Complete results of pairwise comparisons are provided in the Supplementary Materials (Table S3).

Incubation at 37 °C following short-term induction with 10 μM AI shifts the single-cell fluorescence distributions toward lower values, indicating redistribution of the accumulated GFP during cell growth with almost no synthesis of the new protein. At the same time, the distribution becomes narrower: further incubation after interruption of induction leads to a reduction in cell-to-cell variability (Figure 4d).

## 4. Discussion

The pIR-DPAl vector, based on the QS module of the psychrophilic bacterium *A. logei*, was previously described in a 2023 study [18]. In that work, it was shown that biosynthesis of the target protein is initiated upon the appearance of an autoinducer, either endogenous or exogenous, in the medium at 22 °C and is terminated when the temperature is increased to 37 °C. Under the experimental conditions tested, the expression vector enabled accumulation of the target protein up to 33% of the total cellular protein. The ability to terminate induction by raising the temperature to 37 °C made it possible to fix the target protein content in the cell culture, while varying the autoinducer concentration and induction time allowed cultures with the desired level of target protein accumulation to be obtained. However, the uniformity of induction remained unresolved. In particular, it was unclear whether intermediate target protein levels reflected the coexistence of induced and non-induced cell subpopulations or whether induction occurred uniformly across the culture, allowing regulation of target protein content in each individual cell. 

The possibility of non-uniform induction was important to consider because quorum sensing circuits are not necessarily activated synchronously at the single-cell level. In LuxR/LuxI-type systems, autoinducer-dependent activation is coupled to positive feedback through autoinducer synthase expression [24]. Experimental analysis of the *V. fischeri* LuxR/LuxI circuit demonstrated a rapid transition between low- and high-expression states over a narrow autoinducer concentration range. Single-cell studies further support this view: individual *V. fischeri* cells differ markedly in both the timing and intensity of luminescence induction, even at autoinducer concentrations that saturate the light output of the bulk population [17]. Similar heterogeneity has been reported in *Vibrio harveyi* and *Pseudomonas aeruginosa* [25,26]. Together, these observations indicate that intermediate QS-dependent expression levels measured at the culture level may arise either from homogeneous partial induction of all cells or from a changing ratio of induced and non-induced subpopulations. 

In this study, we were focused on investigation of single-cell distribution of reporter protein. We modified the pIR-DPAl plasmid [18] to obtain expression vectors pBMS10 and pBMS10ΔI. The promoter region upstream of target gene was changed, in particular the “−10” sequence has been brought into consensus and the distance between transcription and translation start sites has been reduced. Induction of the system occurs with a decrease in temperature to 22 °C in a dose-dependent manner with 3OxoC6-HSL and stops with an increase in temperature to 37 °C (Figure 2). The pBMS10 vector provides cells with the ability to autoinduce, while the induction of cells with pBMS10ΔI is strictly regulated by the addition of exogenous AI.

Flow cytometry indicated that intermediate expression levels in a cell culture are predominantly achieved through uniform changes in target protein content across all cells, rather than through shifts in the ratio between ON/OFF cell subpopulations, particularly at longer induction times. One possible explanation for the observed expression pattern is the use of freely diffusing autoinducer molecules. Active transport of an inducer, as reported for some conventional inducible systems, may promote heterogeneous intracellular accumulation and population bimodality, whereas passive diffusion is expected to produce a more even distribution of inducer molecules between cells. Indeed, passive diffusion of the autoinducer alone may not be sufficient to guarantee homogeneous QS activation. Phenotypic heterogeneity in QS activation has been reported across diverse species and is not always resolved by providing saturating levels of autoinducer [5,27]. Notably, in *P. aeruginosa*, saturating concentrations of exogenous 3-oxo-C12-HSL fail to shift the *las* QS system to a homogeneously quorate state, because a negative-feedback loop in which LasR coactivates both the *lasI* synthase and its own repressor RsaL actively promotes and sustains cell-to-cell variation in QS activation, independent of AI availability [28]. This contrasts with our observations for the LuxR2/LuxI system studied here, in which no analogous embedded negative-feedback element has been described and in which exogenous AI addition was largely sufficient to drive the population toward a homogeneous expression state after sufficient induction time. This comparison suggests that the presence or absence of additional regulatory feedback loops within a given QS circuit, rather than the diffusible nature of the autoinducer alone, may be a key determinant of whether a QS-based expression system achieves population-wide homogeneity upon induction.

It should be noted, however, that at short induction times, heterogeneity may be observed—some cells did not react to the exogenous AI within 30 min and expression of the target gene in these cells was not detectable. Increasing the cultivation time, regardless of whether induction was interrupted by heating or not, leads to a reduction in cell-to-cell variability and the formation of uniform protein content in cells. No bimodal ON/OFF expression was detected after 180 min.

Comparison of systems with and without the *luxI* gene, under the conditions tested in the study, did not reveal a significant effect of endogenous synthesis of the autoinducer on the heterogeneity of expression. LuxI plays a key role in autoinduction and it should be noted that autoinduction through endogenous AI synthesis reduces the capacity to regulate the level of target protein content in the cell by varying the concentration of exogenous AI (Figure 3).

Increasing temperature results in a strong reduction in target protein synthesis or even switching it off and enables fixing of an intermediate target protein level (Figure 2 and Figure 4). Further incubation results in a decrease in the average amount of target protein per cell and a reduction in the heterogeneity of the system after induction is interrupted. The equalization of distributions observed after stopping induction is likely associated with cell growth and division processes leading to redistribution of the target protein between cells. The absence of the *luxI* gene in the system allows for more precise and predictable fixation of target protein content in the cell. This effect may be particularly useful in tasks requiring the generation of homogeneous populations with a defined expression level. 

QS-regulated expression systems based on Gram-positive bacteria are currently being developed [13,14]. We believe that our results on the uniform regulation of target protein content by changing AI concentration and time of induction at 22 °C, as well as elements of the obtained vectors, will be in demand for the creation of expression systems based on *B. subtilis*.

Our temperature-switchable autoinducer-regulated vectors have practical value beyond fluorescent reporters. The LuxR2/LuxI module was applied for regulating biosynthesis of T7 RNA polymerase in this work and antirestriction proteins of the ArdA/ArdB family in [19,20,21]. ArdA/ArdB DNA-mimicking and DNA-binding proteins inhibit bacterial restriction–modification systems and even could regulate gene expression [29]. Their prolonged, uncontrolled overexpression can be toxic and can interfere with normal host DNA metabolism, while comparing the antirestriction potency of different homologs requires a defined, non-saturating expression level, which could be achieved by using our expression vectors. However, further validation with additional target proteins, higher expression burdens, and scaled-up culture conditions remains an important direction for future work.

## 5. Conclusions

In this study, we characterized at single-cell resolution a temperature-switchable, QS-regulated expression system based on LuxR2/LuxI regulatory elements from the psychrophilic bacterium *A. logei*. *luxR2/luxI* genes from *A. logei* allowed AI-dose-dependent regulation of target gene expression in *E. coli* cells. After sufficient induction time, the formation of distinct ON/OFF subpopulations was not observed and expression occurred rather uniformly both at low and high concentrations of AI. Some heterogeneity was observed shortly after induction started, but further incubation either with continued induction at 22 °C or interrupted induction at 37 °C narrowed cell-to-cell variation of target protein content. The autoinducer synthase LuxI enables autoinduction but it reduces the precision with which target protein levels can be controlled via exogenous AI. Under the tested conditions, we did not observe a substantial effect of *luxI* on expression homogeneity.

The developed system, characterized here primarily using the sfGFP reporter and additionally validated with T7 RNA polymerase, represents a promising tool for biotechnological applications. 

## Data Availability

The original contributions presented in this study are included in the article/supplementary material. Further inquiries can be directed to the corresponding author.

## Supplementary Materials

The following supporting information can be downloaded at: https://www.mdpi.com/article/10.3390/biotech15030057/s1, Figure S1: OD600 growth curves of TG1 pBMS10-sfGFP cultures induced with 10 μM AI, corresponding to the two conditions: cells incubated at 22 °C for 30 minutes and then shifted to 37 °C (interrupted induction), and cells maintained continuously at 22 °C without a temperature shift (non-interrupted induction); Figure S2. SDS–PAGE analysis of T7 RNA polymerase expression; Figure S3. SDS–PAGE analysis of sfGFP accumulation following induction at 22 °C and interruption of induction by temperature shift to 37 °C in the presence of 10 μM exogenous AI; Figure S4: Distributions of sfGFP fluorescence in TG1 pBMS10-sfGFP cells at different concentrations of autoinducer and induction for 30 min and 180 min in triplicate (Rep 1, Rep 2, Rep 3); Figure S5: Distributions of sfGFP fluorescence in TG1 pBMS10ΔI-sfGFP cells at different concentrations of autoinducer and induction for 30 min and 180 min in triplicate (Rep 1, Rep 2, Rep 3); Figure S6: Fluorescence microscopy of TG1 pBMS10-sfGFP cells after 3 h of induction with 10 nM (a) and 10 μM (b) AI; Figure S7: Comparison of sfGFP fluorescence distributions measured immediately after 30 min of induction and after subsequent incubation at 37°C for 2.5 hours in TG1 pBMS10-sfGFP and TG1 pBMS10ΔI-sfGFP cells with the addition of 10 uM autoinducer in triplicate (Rep 1, Rep 2, Rep 3); Table S1: Primers used in the study; Table S2: Statistical analysis of AI- and time-dependent responses in TG1 pBMS10-sfGFP and TG1 pBMS10ΔI-sfGFP; Table S3: Statistical analysis of fluorescence and variability following interrupted induction.

## Abbreviations

The following abbreviations are used in this manuscript:

QS	quorum sensing
AI	autoinducer
3OxoC6-HSL	N-(3-oxohexanoyl)-L-homoserine lactone
rCV	robust relative coefficient of variation
MAD	median absolute deviation
AHL	acyl-homoserine lactone
