# Supplementary material for: Psychrophilic Quorum Sensing Genes Enable Unimodal, Adjustable Protein Expression Across the Entire Escherichia coli Population
Source: BioTech (Basel). 2026 Jul 21;15(3):57. doi: 10.3390/biotech15030057 (PMC13398223; doi:10.3390/biotech15030057)
Supplement: Supplementary file 1 [file biotech-15-00057-s001.zip › biotech-4386672-supplementary.pdf]

Psychrophilic Quorum Sensing Genes Enable Unimodal, Adjustable Protein Expression Across the Entire *Escherichia coli* Population

Table S1. Primers used in the study.

Primer	Sequence, 5′->3′
TATAGT-Rev	AAATGACTAATTTATAATTTTAGAAAAC-TATAGCAGGGTA
PluxCshort-Dir	TTTTCTAAAATTATAAATTAGTCATTTAA-TAAAAAATTTAACA
sfGFPdir-pMBS	CATTATTTTAAATATATTAA-TAAGGAGGTATCATTATGTCAAAAGGAGAA-GAACTTTTTACAGGT
sfGFPrev-pMBS	AATGGTGGTGATGGTGATGCATTATTTATAAAGT TCGTCCATACCGTGA
pBMS-DluxIrev	CCTTTTTAAATTGTTCATTAATCGGTTAACTGAC-TATAGCAGGCAAATTTCC
pBMS-DluxIdir	CCGATTAATGAACAATTTAAAAAGGCTGTATT
T7_pIRDAl_dir	ATCACCATCACCACCATTCCGGAATGAACAC-GATTAACATCGCTAAGAACG
T7_pIRDPAl_rev	AGTTATTGCTCAGCGGTGTCATTATTTAC-GCGAACGCGAAGTCC

Table S2. Statistical analysis of AI- and time-dependent responses in TG1 pBMS10-sfGFP and TG1 pBMS10ΔI-sfGFP.

Response variables: median log_{10}-transformed GFP fluorescence and robust coefficient of variation (rCV). Model: response ~ luxI × concentration × time + (1 ∣ colony).

(a) Type III ANOVA

Effect	Median log10 GFP			rCV		
**Effect**	**F**	**df**	**p**	F	df	p
luxI	2.50	1, 4	0.189	0.02	1, 4	0.891
concentration	182.61	2, 20	1.4×10^{-13}	19.89	2, 20	1.8×10^{-5}
time	299.75	1, 20	1.7×10^{-13}	5.76	1, 20	0.026
luxI × concentration	3.26	2, 20	0.059	5.24	2, 20	0.015
luxI × time	12.53	1, 20	0.0021	0.84	1, 20	0.371
concentration × time	41.00	2, 20	8.4×10^{-8}	55.44	2, 20	6.9×10^{-9}
luxI × concentration × time	1.66	2, 20	0.215	2.66	2, 20	0.094

(b) Post hoc pairwise comparisons of AI concentration within each *luxI* × time group.

Response	luxI	Time	Comparison	Estimate	t	p (Holm)
median log10	present	30 min	0 nM – 10 nM	−0.111	−0.627	0.537
median log10	present	30 min	0 nM – 10 µM	−0.973	−5.491	<0.0001
median log10	present	30 min	10 nM – 10 µM	−0.861	−4.864	0.0002
median log10	absent	30 min	0 nM – 10 nM	−0.181	−1.019	0.320
median log10	absent	30 min	0 nM – 10 µM	−1.108	−6.256	<0.0001
median log10	absent	30 min	10 nM – 10 µM	−0.927	−5.237	<0.0001
median log10	present	180 min	0 nM – 10 nM	−1.515	−8.553	<0.0001
median log10	present	180 min	0 nM – 10 µM	−1.972	−11.134	<0.0001
median log10	present	180 min	10 nM – 10 µM	−0.457	−2.581	0.0178
median log10	absent	180 min	0 nM – 10 nM	−1.699	−9.591	<0.0001
median log10	absent	180 min	0 nM – 10 µM	−2.715	−15.332	<0.0001
median log10	absent	180 min	10 nM – 10 µM	−1.017	−5.741	<0.0001
rCV	present	30 min	0 nM – 10 nM	−0.098	−1.257	0.223
rCV	present	30 min	0 nM – 10 µM	−0.592	−7.591	<0.0001
rCV	present	30 min	10 nM – 10 µM	−0.494	−6.333	<0.0001
rCV	absent	30 min	0 nM – 10 nM	−0.171	−2.196	0.040
rCV	absent	30 min	0 nM – 10 µM	−0.599	−7.677	<0.0001
rCV	absent	30 min	10 nM – 10 µM	−0.428	−5.481	<0.0001
rCV	present	180 min	0 nM – 10 nM	0.062	0.790	0.439
rCV	present	180 min	0 nM – 10 µM	0.217	2.775	0.035
rCV	present	180 min	10 nM – 10 µM	0.155	1.985	0.122

Response	luxI	Time	Comparison	Estimate	t	p (Holm)
rCV	absent	180 min	0 nM − 10 nM	−0.369	−4.735	0.0004
rCV	absent	180 min	0 nM − 10 µM	−0.004	−0.055	0.957
rCV	absent	180 min	10 nM − 10 µM	0.365	4.680	0.0004

(c) Post hoc comparisons of presence *luxI* within each concentration × time group.

Response	Concentration	Time	Estimate	SE	t	p (Holm)
median log10	0 nM	30 min	0.011	0.205	0.054	0.958
median log10	10 nM	30 min	−0.058	0.205	−0.285	0.779
median log10	10 µM	30 min	−0.124	0.205	−0.607	0.551
median log10	0 nM	180 min	0.764	0.205	3.728	0.0015
median log10	10 nM	180 min	0.580	0.205	2.831	0.0110
median log10	10 µM	180 min	0.020	0.205	0.099	0.922
rCV	0 nM	30 min	0.002	0.078	0.028	0.978
rCV	10 nM	30 min	−0.071	0.078	−0.911	0.372
rCV	10 µM	30 min	−0.005	0.078	−0.059	0.954
rCV	0 nM	180 min	0.251	0.078	3.219	0.0037
rCV	10 nM	180 min	−0.180	0.078	−2.307	0.030
rCV	10 µM	180 min	0.030	0.078	0.389	0.701

Table S3. Statistical analysis of fluorescence and variability following interrupted induction.

Model: response ~ strain × induction state + (1 ∣ colony), with three induction states: no induction (0 µM AI), low induction (10 µM AI, 30 min), and interrupted induction (10 µM AI, 30 min followed by 2.5 h at 37 °C).

(a) Type III ANOVA

Effect.	Median log10 GFP			rCV		
Effect	F	df	p	F	df	p
strain (luxI)	0.030	1, 4	0.870	0.147	1, 4	0.721
induction state	517.20	2, 8	3.5×10^{-9}	445.63	2, 8	6.3×10^{-9}
strain × induction state	0.002	2, 8	0.998	1.51	2, 8	0.279

(b) Post hoc pairwise comparisons of induction states (Holm-corrected).

Response	Strain	Comparison	Estimate	t	p (Holm)
median log10	+luxI	low − no induction	1.106	22.082	<0.0001
median log10	+luxI	interrupted − no induction	0.784	15.654	<0.0001
median log10	+luxI	low − interrupted	0.322	6.427	0.0002
median log10	−luxI	low − no induction	1.108	22.116	<0.0001
median log10	−luxI	interrupted − no induction	0.789	15.747	<0.0001
median log10	−luxI	low − interrupted	0.319	6.369	0.0002
rCV	+luxI	low − no induction	0.616	21.390	<0.0001
rCV	+luxI	interrupted − no induction	0.300	10.418	<0.0001
rCV	+luxI	low − interrupted	0.316	10.972	<0.0001
rCV	−luxI	low − no induction	0.599	20.794	<0.0001
rCV	−luxI	interrupted − no induction	0.351	12.182	<0.0001
rCV	−luxI	low − interrupted	0.248	8.612	<0.0001

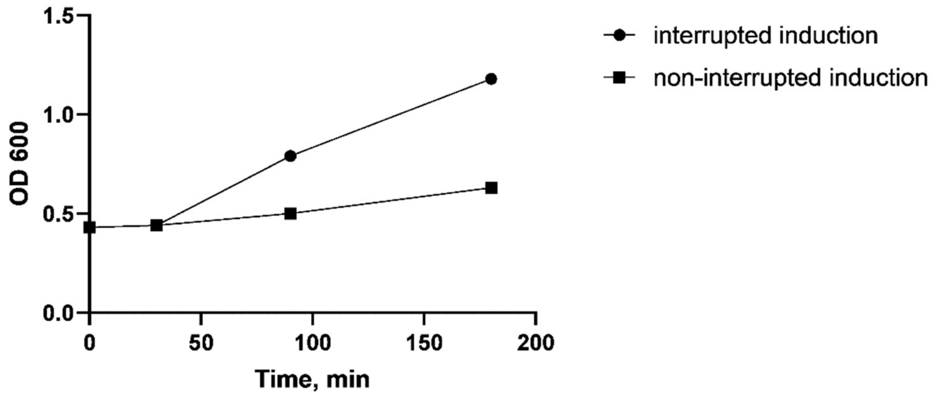

Figure S1. OD600 growth curves of TG1 pBMS10-sfGFP cultures induced with 10 μM AI, corresponding to the two conditions: cells incubated at 22 °C for 30 minutes and then shifted to 37 °C (interrupted induction), and cells maintained continuously at 22 °C without a temperature shift (non-interrupted induction).

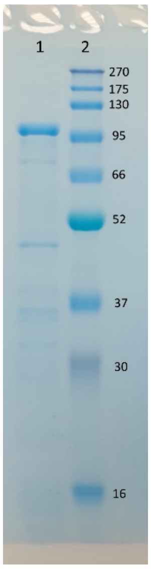

Figure S2. SDS–PAGE analysis of T7 RNA polymerase expression. 1 - TG1 pBMS10-T7, 19h of induction at 22 °C. 2 – protein molecular weight marker Rav10 (Biolabmix, Russia).

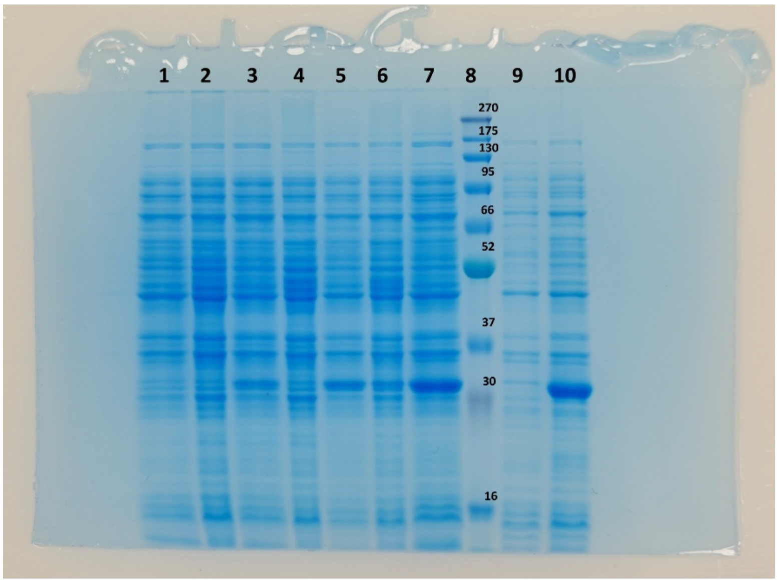

Figure S3. SDS–PAGE analysis of sfGFP accumulation following induction at 22 °C and interruption of induction by temperature shift to 37 °C in the presence of 10 μM exogenous AI. TG1 pBMS10-sfGFP: 1 - before induction, 2 – 180 min at 37 °C, 3 – 30 min at 22 °C, 4 - 30 min at 22 °C followed by incubation at 37 °C for 150 min, 5 - 90 min at 22 °C, 6 - 90 min at 22 °C followed by incubation at 37 °C for 90 min, 7 - 180 min at 22 °C, 9 – 19 h at 37 °C, 10 – 19 h at 22 °C, 8 - protein molecular weight marker Rav10 (Biolabmix, Russia).

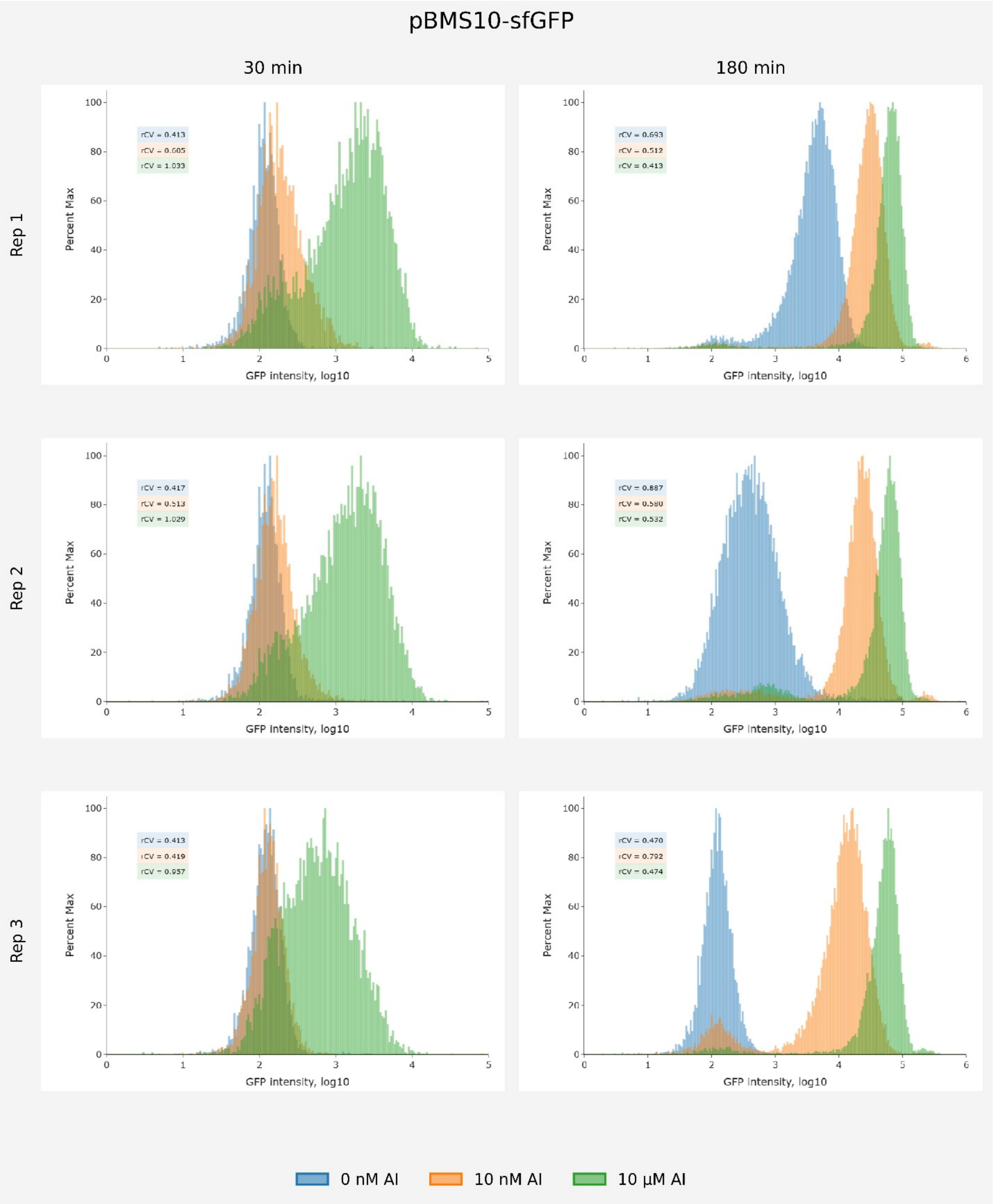

Figure S4. Distributions of sfGFP fluorescence in TG1 pBMS10-sfGFP cells at different concentrations of autoinducer and induction for 30 min and 180 min in triplicate (Rep 1, Rep 2, Rep 3).

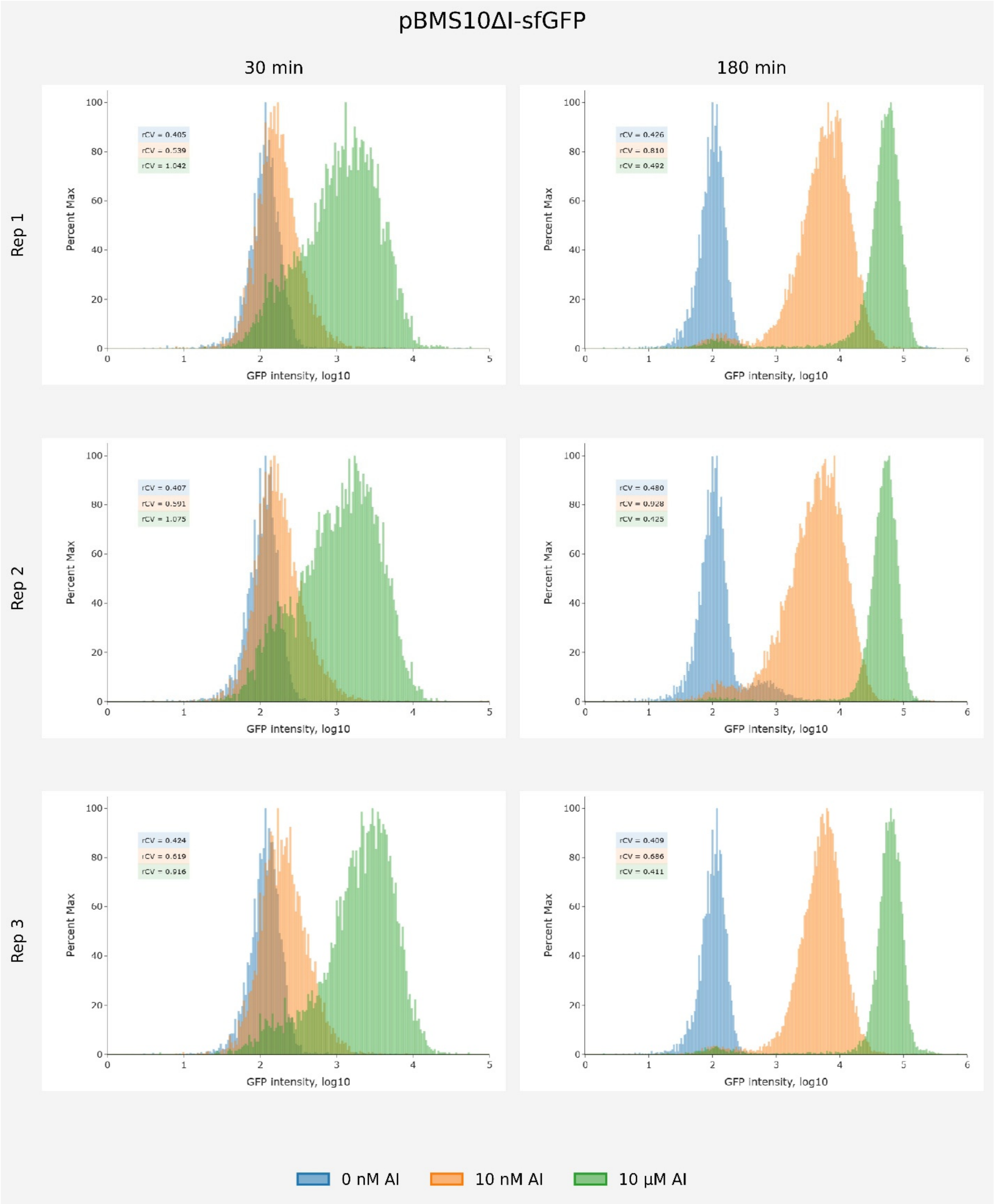

Figure S5. Distributions of sfGFP fluorescence in TG1 pBMS10ΔI-sfGFP cells at different concentrations of autoinducer and induction for 30 min and 180 min in triplicate (Rep 1, Rep 2, Rep 3).

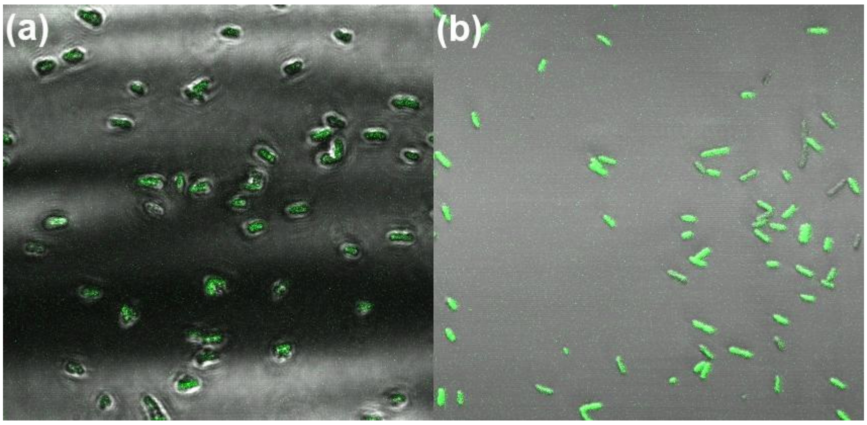

Figure S6. Fluorescence microscopy of TG1 pBMS10-sfGFP cells after 3 h of induction with 10 nM (a) and 10 μM (b) AI. Images were obtained using an overlay of T-PMT (transmitted light photo-multiplier, black and white display) and channel mode (490-540 nm range, 488 nm laser excitation, green display).

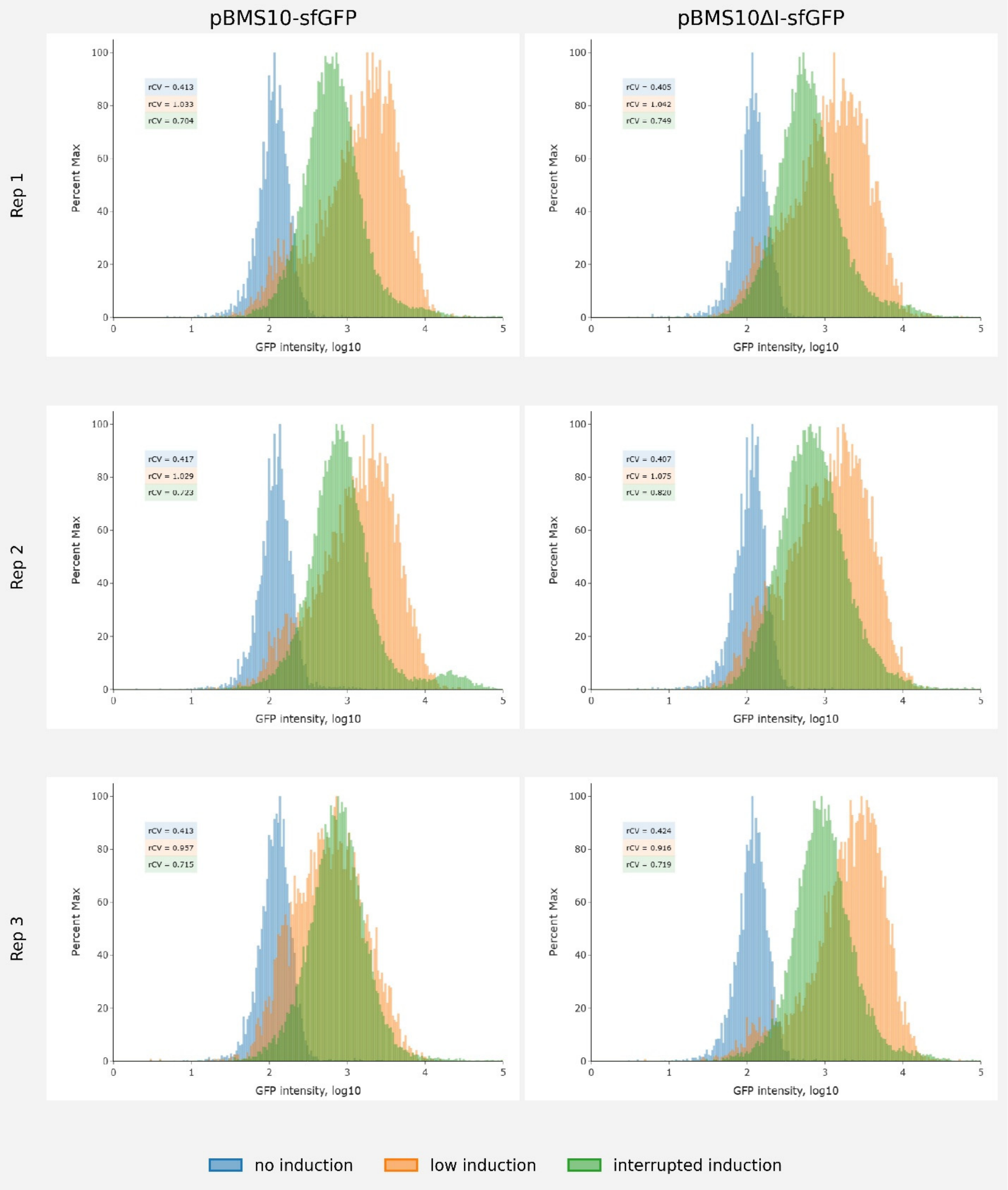

Figure S7. Comparison of sfGFP fluorescence distributions measured immediately after 30 min of induction and after subsequent incubation at 37°C for 2.5 hours in TG1 pBMS10-sfGFP and TG1 pBMS10ΔI-sfGFP cells with the addition of 10 uM autoinducer in triplicate (Rep 1, Rep 2, Rep 3). Fluorescence distributions without induction are shown as a control. No induction – 0 nM AI, 30 min at 22°C; low induction – 10 μM, 30 min at 22°C; interrupted induction - 10 μM, 30 min at 22°C, 150 min at 37°C.
